# Relevance Feedback Based Query Expansion Model Using Borda Count and Semantic Similarity Approach

**DOI:** 10.1155/2015/568197

**Published:** 2015-12-07

**Authors:** Jagendra Singh, Aditi Sharan

**Affiliations:** School of Computer and Systems Sciences, Jawaharlal Nehru University, New Delhi 110067, India

## Abstract

Pseudo-Relevance Feedback (PRF) is a well-known method of query expansion for improving the performance of information retrieval systems. All the terms of PRF documents are not important for expanding the user query. Therefore selection of proper expansion term is very important for improving system performance. Individual query expansion terms selection methods have been widely investigated for improving its performance. Every individual expansion term selection method has its own weaknesses and strengths. To overcome the weaknesses and to utilize the strengths of the individual method, we used multiple terms selection methods together. In this paper, first the possibility of improving the overall performance using individual query expansion terms selection methods has been explored. Second, Borda count rank aggregation approach is used for combining multiple query expansion terms selection methods. Third, the semantic similarity approach is used to select semantically similar terms with the query after applying Borda count ranks combining approach. Our experimental results demonstrated that our proposed approaches achieved a significant improvement over individual terms selection method and related state-of-the-art methods.

## 1. Introduction

Retrieving relevant documents that can fulfill user need is one of the major challenges in the information retrieval (IR) system. One of the most feasible and successful techniques to handle this problem is PRF based query expansion (QE), where some top documents retrieved in the first iteration are used to expand the original user query. To consider the above problem, there is a need of automatic PRF based QE techniques that can automatically reformulate the original user query. In some last years, it has been observed that the volume of data available online has dramatically increased while the number of query terms searched remained very less. According to the authors [1], the average query length was 2.30 words, the same about query length has been reported ten years after by Rijsbergen [2]. While there has been a slight increase in the number of long queries (of five or more words), the most prevalent queries are still those of one, two, and three words. In this situation, the need and the scope for automatic query expansion (AQE) have increased, but it has some problems.

The main problem of AQE is that it cannot work efficiently due to the inherent sparseness of the user query terms in the high dimensional corpus. Another problem is that not all the terms of top retrieved documents (feedback documents) are important for the QE. Some of the QE terms may be redundant or irrelevant. Some may even misguide the result, especially when there are more irrelevant QE terms than relevant ones. QE selection aims to remove redundant and irrelevant terms from the term pool (top retrieved documents as feedback documents for selecting user QE terms), and the selected QE terms set should contain sufficient and reliable information about the original document. Thus, QE terms selection should not only reduce the high dimensionality of the feedback document corpus (term pool), but also provide a better understanding of the documents, in order to improve the AQE result. Feedback based different QE terms selection methods have been widely used in AQE, and it has been reported that QE terms selection methods can improve the efficiency and accuracy of IR model.

Traditional QE terms selection methods for AQE are either corpus statistics based or term association based, depending on the used algorithm in the retrieval model. Term association based terms selection methods, such as mutual information [3] and cooccurrence information [2, 4], estimate the goodness of each term based on the occurrence of terms in feedback documents (term pool). Corpus statistics based QE terms selection methods, such as Kullback-Leibler Divergence [5], information gain [6], and Robertson selection value [7], estimate the goodness of each term based on the distribution of terms across the corpus.

Most of study on the QE terms selection focused on the performance improvement of individual QE terms selection methods. However, it remains as a challenge to develop an individual QE terms selection method that would outperform other methods in most cases. Moreover, as multiple QE terms selection methods are available, it is natural to combine them for better performance by taking advantage of their individual strength. In the past, experiments of combining multiple query terms selection methods have been conducted, but no theoretical analysis has been done. Combinations of two uncorrelated and high-performing QE terms selection methods have been tested [8]. The author in [5] developed a naive combination of cooccurrence and probabilistic kinds of method, with which the developed approach obtained results that improve those obtained with any of them separately. This result confirms that the information provided by each approach is of a different nature and, therefore, can be used in a combined manner. The author in [9] discussed various ranked search results aggregation methods such as Borda and Condorcet and confirmed the improvement in search quality.

A set of expansion terms are obtained after applying Borda count based ranks combining approach, we analyzed that some obtained expansion terms are not related to user query semantically. Therefore, it became compulsory to check the semantic meaning of selected expansion terms with the user query to avoid query drifting problem. For this purpose, we use the concept of semantic similarity with the help of WordNet. In the literature survey, semantics based approaches are also used in some research; for example, concept level approach is used in sentiment analysis [10], representation of word and phrase [11], finding patterns for concept level sentiment analysis [12], and analysis of emotions in natural language [13]. Some other works have been done by using the concept of semantic similarity for IR with QE. The authors in [14, 15] proposed a QE technique using WordNet lexical chains, hypernym/hyponymy, and synonymy relations in WordNet. Lexical chains are used as the basic expansion rules and confirmed that QE can improve the query performance dramatically. In [16] Liu et al. explained the use of WordNet lexical ontology for both expanding query and selecting proper sense of expansion terms and achieved reasonable performance improvement. After applying semantic filtering, a refined set of additional expansion terms was obtained. After that, an approach of reweighting was required that will provide higher weight to original query terms than the additional expansion terms. The authors in [17] present a new method for query reweighting to deal with document retrieval. It reweights a user's query vector, based on the user's relevance feedback, to improve the performance of document retrieval systems.

The experiments of the proposed model are performed on two well-known benchmark datasets, namely, FIRE and TREC-3. For performance evaluation, the proposed model has been compared with Okapi-BM25 [18] and Aguera and Araujo's model [5]. The proposed methods increase the precision rates and the recall rates of IR systems for dealing with document retrieval. It also gets a significant higher average recall rate, average precision rate, and *F*-measure on both datasets.

The major contributions of this work are summarized as follows:

(1) First, we present IG, cooccurrence, KLD, and RSV terms selection methods for PRF based AQE; with this, the experimental analyses of all these terms selection methods are presented with evaluation parameter score.
(2) Second, we propose most popular Borda rank aggregation methods for combining different ranked lists of expansion terms selected by IG, cooccurrence, KLD, and RSV methods discussed in step (1).
(3) Third, we propose semantic similarity approach to filter out the irrelevant and redundant expansion terms with context to user query obtained from step (2). Next, additional expansion terms were used after applying the reweighting approach.
(4) Finally, paired *t*-test is conducted between our proposed approaches and the other model considered as the baseline model.

The organization of this paper is as follows. In Section 2, we briefly introduce PRF based document selection and four individual QE terms selection methods.  Section 3 explained our proposed model and its algorithm with Borda count based ranking approach and semantic similarity based approach.  Section 4 presents the experimental results of different QE terms selection methods and then compared them with each other; next in this section our proposed approaches results are presented and compared or analyzed with baseline approaches in terms of the precision, recall, and *F*-measure on both FIRE and TREC datasets. Finally, Section 5 presents the conclusion and future research directions.

## 2. PRF Document Selection and Query Expansion Terms Selection Methods

In this section, we briefly discuss PRF based QE. In Section 2.1, we discuss the criteria for selecting the relevant documents that provide a source for selecting candidate expansion terms. In the rest of Sections 2.2–2.5, we explain four QE terms selection methods. It requires two parameters to be set: value of top number of relevant documents to be used for selecting candidate expansion terms and number of expansion terms to be used for expanding the query. The settings of these parameters have to be done empirically.

### 2.1. PRF Based Top Documents and Terms Selection

**Figure 1:**
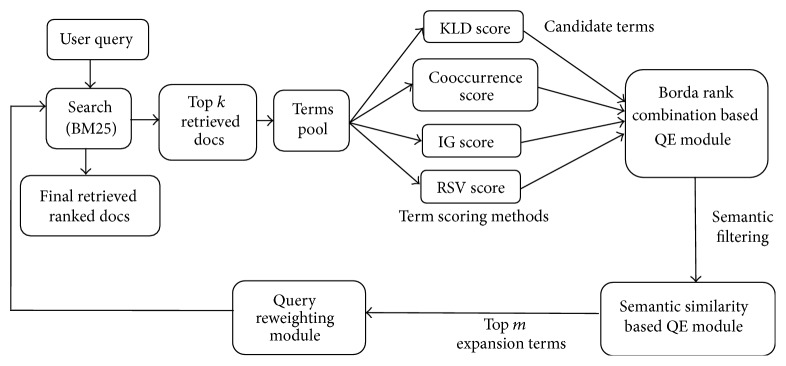
The diagram of the proposed AQE model.

**Figure 2:**
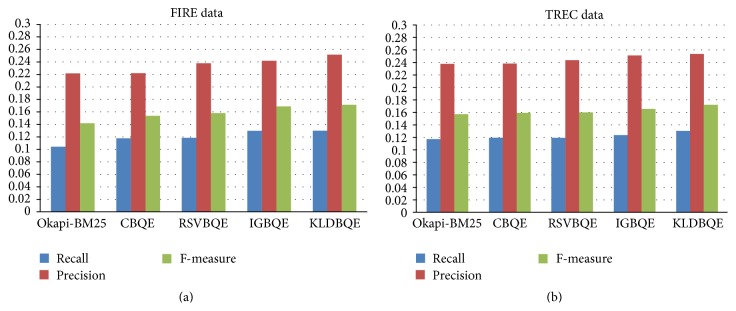
Recall, precision, and *F*-measures values of all individual approaches on both FIRE and TREC dataset (for top 10 retrieved documents, discussed in Tables 2 and 3).

We have used an efficient Okapi-BM25 similarity measure for selecting an initial set of retrieved documents, which is more efficient than the traditionally used Cosine similarity measure. Figures 1 and 2 show the architecture of our proposed AQE retrieval model based on rank aggregation and semantic filtering schemes. To construct the term pool, we first retrieve a number of top documents from first retrieved documents for the query using a matching function. In our problem, we used an Okapi-BM25 matching function to retrieve first relevant documents. The Okapi-BM25 measure is given by following [18]

(1) $$\begin{array}{c} \text{Okapi}\left(\;Q,D_{i}\;\right)=\underset{t\in Q\cap D_{i}}{\sum }w\frac{\left(k_{1}+1\right)\text{tf}}{K+\text{tf}}\frac{\left(k_{3}+1\right)\text{qtf}}{k_{3}+\text{qtf}}, \end{array}$$

where *Q* is the query that contains terms, tf is the term frequency of term *t* in the *i*th document *D*_*i*_, and qtf is the term frequency in query *Q*. Next, *k*_1_, *b*, and *k*_3_ are constant parameters, the values of parameters that we used in our experiments are based on the Robertson et al. [18] (*k*_1_ = 1.2, *b* = 0.75, and *k*_3_ = 7.0):

(2) K=k11−b+b·dlavdl,w=log⁡N−n+0.5n+0.5,

where *N* is the number of documents and *n* is the number of documents containing the term *t*. Parameters dl and avdl are document length and average document length. Once the top relevant document was retrieved with the help of Okapi-BM25 method discussed in this section, all the unique terms of top documents are selected to form term pool or candidate terms set denoted by Tp. The terms are ranked by any of the several scoring techniques/measures available to rank the terms on the basis of their appropriateness for expansion. These scoring measures are presented in coming subsections.

### 2.2. Kullback-Leibler Divergence Based Query Expansion

The Kullback-Leibler Divergence (KLD) [5] is well-known in information theory [4]. KLD based approach has been used in natural language and speech processing applications based on statistical language modeling in IR (Robertson, 1990). KLD can be used as a term scoring function that is based on the differences between the distribution of terms in the collection of top retrieved relevant documents and entire document collection. Thus, the following equation can be used to find the KLD score of candidate expansion term:

(3) $$\begin{array}{c} \text{KLD}\left(\;t\;\right)=P_{R}\left(\;t\;\right)\log\frac{P_{R}\left(t\right)}{P_{C}\left(t\right)}, \end{array}$$

where *P*_*R*_(*t*) is the probability of presence of term *t* in top retrieved document collection *R*, given by

(4) $$\begin{array}{c} P_{R}\left(\;t\;\right)=\frac{\sum_{d\in R}\text{tf}\left(t/d\right)}{\sum_{d\in R}\sum_{t\in d}\text{tf}\left(t/d\right)}. \end{array}$$

And *P*_*C*_(*t*) is the probability of presence of term *t* in the entire document collection *C*, explained by

(5) $$\begin{array}{c} P_{C}\left(\;t\;\right)=\frac{\sum_{d\in C}\text{tf}\left(t/d\right)}{\sum_{d\in C}\sum_{t\in d}\text{tf}\left(t/d\right)}. \end{array}$$

Equation (3) is used to find the KLD score of candidate expansion terms. Some top scored candidate terms are used to expand the user original query. This type of query expansion is called KLD based query expansion (KLDBQE).

### 2.3. Cooccurrence Based Query Expansion

The most feasible method for selecting the QE terms is to initially score the terms on the basis of their cooccurrence with original user query terms. The concept of term cooccurrence has been used since the 90s for identifying some kind of relationship among terms in the documents set [4]. According to van Rijsbergen [2], the idea of using cooccurrence statistics is to find the relationship between document corpus and the query terms, and the author used this idea to expand the original user queries.

We can use co⁡(*t*_*i*_, *t*_*j*_) to quantify the strength of cooccurrence based association between two terms. Following are some well-known cooccurrence coefficient methods; here co⁡(*t*_*i*_, *t*_*j*_) can be given by one of the following equations:

(6) Jaccardti,tj=cijci+cj−cij,diceti,tj=2cijci+cj,Cosineti,tj=cijcicj,

where *c*_*i*_ and *c*_*j*_ are the number of documents that contain terms *t*_*i*_ and *t*_*j*_, respectively, and *c*_*ij*_ is the document numbers that contain both terms *t*_*i*_ and *t*_*j*_ together.

We can use these cooccurrence coefficient values to find the value of similarity between user query terms *q*_*i*_ and the candidate expansion term *c*. But there is a problem of query drifting by adding these high similar terms with the user query terms. For handling this kind of problem, one can use the concept of inverse document frequency (idf). With the help of candidate term idf value and normalization cooccurrence coefficient value with user query terms, the codegree coefficient of the candidate term is obtained, explained in (8). Consider

(7) Codegreeqi,c=log10⁡co⁡qi,c+1·idfclog10⁡D,

(8) idfc=log10⁡NNc,

where *N* is the number of documents in the corpus, *D* is the number of top ranked retrieved documents considered, *q*_*i*_ is the *i*th query term, *c* is the candidate expansion term, and *N*_*c*_ is the number of documents in the corpus that contain term *c*. And co⁡(*q*_*i*_, *c*) is the number of cooccurrences between *q*_*i*_ and *c* in the top ranked documents, that is, Jaccard(*q*_*i*_, *c*).

Equation (8) can be used for finding similarity of a term *c* with individual query term *q*_*i*_. To obtain a value measuring how well *c* is for the whole query *Q*, there is a need to combine its codegree with all individual original query terms present in query. So we use

(9) $$\begin{array}{c} {\text{Cooccurrence}}_{\text{final}}\left(\;Q,c\;\right)=\underset{q_{i}\text{ in }Q}{\prod }\left(\;\text{Codegree}\left(\;q_{i},c\;\right)\;\right). \end{array}$$

Finally, (9) is used to find the cooccurrence coefficient score of candidate expansion terms. This type of query expansion is called Cooccurrence Based Query Expansion (CBQE).

### 2.4. Information Gain Based Query Expansion (IGBQE)

Information gain (IG) coefficient is a parameter to find the degree of class prediction by the presence or absence of a term in a documents set [6]. Let *C* = {*c*_1_, *c*_2_,…, *c*_|*c*|_} be the set of classes; in our case there are two classes: first, set of initially retrieved relevant documents for a user query called PRF documents; second, set of nonrelevant documents for the same query. Now the value of information gain coefficient of a term *t* can be explained as follows:

(10) IGt=−∑i=1CPcilog⁡Pci+Pt∑i=1CPci ∣ tlog⁡Pci ∣ t+Pt−∑i=1CPci ∣ t−log⁡Pci ∣ t−,

where *P*(*t*) is the probability that term *t* occurs, $\overset{-}{t}$ means that term *t* does not occur (i.e., $P\left(\overset{-}{t}\right)=1-P(t)$), *P*(*c*_*i*_) is the probability of the *i*th class value, *P*(*c*_*i*_∣*t*) is the conditional probability of the *i*th class value given that *t* occurs, and $P\left(c_{i}|\overset{-}{t}\right)$ is the conditional probability of the *i*th class value given that *t* does not occur. The value of information gain coefficient is used to measure the importance of a term with respect to all the classes. The terms of term pool or top retrieved feedback document are ranked based on the value obtained from (10). Some high IG scored candidate terms selected for expanding the user query. This type of query expansion is called IG Based Query Expansion (IGBQE).

### 2.5. Robertson Selection Value Based Query Expansion

The RSV method [7] is based on Swets model of IR system performance [19]. The system is assumed to retrieve items by ranking them according to some measure of association with the query. The principle idea of the Swets theory is to examine the distribution of values of this match function over the document collection. More specifically, it considers two such distributions, one for the relevant documents and one for the nonrelevant ones. If the retrieval system is any good, the two distributions will be different; in particular the match function values will generally be higher for relevant documents than for nonrelevant ones.

In general, the more the two distributions are separated, the better the performance of the system will be. Other things being equal, the higher the difference *d* = *μ*_*R*_ − *μ*_*N*_ between the means of the two distributions, the better the performance. Actually the measure of performance proposed by Swets and an alternative proposed by Brookes [20] can both be expressed as *d* normalized by some function of standard deviations of the distributions. However, these measures are associated with the assumption that the distributions are normal. This would not be an appropriate assumption for the present situation. So the present argument is based on the use of *d*, unnormalized, as a simple measure of performance.

If the weight of candidate term is *W*_*t*_ then those classes that contain the term will have *W*_*t*_ added to their match function values. For the case of query expansion, we consider the candidate term *t* with weight *W*_*t*_. The new mean of relevant and nonrelevant document class is given by *μ*_*R*_ and *μ*_*N*_, respectively.

If *P*_tr_ and *P*_tnr_ correspond to the probability of terms present in relevant and nonrelevant document collection, respectively, the equation for *μ*_*R*_ (mean of relevant document) is given as follows:

(11) $$\begin{array}{c} \left(\;1-P_{\mathrm{tr}}\;\right)\mu_{R}+P_{\mathrm{tr}}\left(\;\mu_{R}+W_{t}\;\right)=\mu_{R}+P_{\mathrm{tr}}W_{t}. \end{array}$$

Similarly, the new mean for *μ*_*N*_ (the nonrelevant documents) is given as follows:

(12) $$\begin{array}{c} \mu_{N}+P_{\text{tnr}}W_{t}. \end{array}$$

And the effectiveness *d*′ is defined as

(13) d′=μR+Ptr⁡Wt−μN−PtnrWt=μR−μN+WtPtr⁡−Ptnr=d+WtPtr⁡−Ptnr.

If differences between two distributions are very low then

(14) $$\begin{array}{c} d^{\prime }=W_{t}\left(\;P_{\mathrm{tr}}-P_{\text{tnr}}\;\right), \end{array}$$

where *d* is the original difference of *μ*_*R*_ and *μ*_*N*_.

Finally, the weight of candidate expansion term is given as follows:

(15) $$\begin{array}{c} =\underset{t\in d}{\sum }w\left(\;t,d\;\right)\left(\;P_{\mathrm{tr}}-P_{\text{tnr}}\;\right), \end{array}$$

where *P*_tr⁡_ is the probability of expansion term in relevant documents and *P*_tnr_ is the probability of expansion term in nonrelevant document or corpus. Equation (15) can be used to find the RSV score of candidate expansion terms. Some top scored candidate terms are used to expand the user original query. This type of query expansion is called RSV Based Query Expansion (RSVBQE).

## 3. Proposed Borda and Semantic Similarity Based Model

Our proposed work can be categorized mainly in two parts:

(i) First, score combination of different individual approaches using Borda count approach.
(ii) Second, apply semantic similarity approach for removing noisy or irrelevant terms.

PRF based QE methods select the candidate terms for expanding the user query from initially retrieved set of documents. We have used an efficient Okapi-BM25 similarity measure for selecting initial set of retrieved documents, which is more efficient then the traditionally used Cosine similarity measure.  Figure 1 shows architecture of our proposed AQE retrieval model based on Borda count rank combination and semantic similarity approaches.

Initially, we use cooccurrence approach, in which words present around the query term in top feedback documents are used for selecting expansion terms; we call it CBQE. In this approach, high cooccurrence value terms selected from cooccurrence approach form a term pool of candidate terms. Further, the information gain method is used to score terms of term pool and some high scored terms are used as query expansion terms; this is called IGBQE. Next, the concepts behind the Kullback-Leibler Divergence (KLD) and Robertson selection value (RSV) are used to score the term pool terms and high scored terms used for expanding the user original query; these QE methods are called KLDBQE and RSVBQE.

Further, well-known Borda count ranks combining scheme is used to combine multiple terms ranks obtained from cooccurrence, IG, KLD, and RSV methods. This rank aggregation method produces a single combined list of candidate terms with their Borda score that is high to low from top to bottom. Top *n* candidate terms, selected from this approach, are used to expand user query: this is called Borda based query expansion (BBQE). The set of candidate terms obtained after applying Borda rank aggregation methods contains some noisy or semantically irrelevant terms with the query. If we include these noisy terms in the process of query expansion, it may lead to the problem of query drifting. Therefore the concept of semantic similarity is used to filter out semantically irrelevant terms obtained from BBQE for query reformulation or expansion that is called Borda and semantic based query expansion (BSBQE). Finally, reformulated query with reweighted expansion terms submitted to the searching engine a list of ranked documents retrieved as a final result for the user query.

### 3.1. Algorithmic Description of the Proposed Model

The sequences of algorithmic steps used during the implementation of the proposed model are defined in Algorithm 1.

**Algorithm 1 (developed for the proposed AQE model).**

 (1) Apply Okapi-BM25 similarity function for retrieving the ranked relevant document with respect to a user query.
 (2) All the unique terms of top *n* retrieved documents obtained from step (1) are selected to form term pool.
 (3) The different method is used to score the unique terms of term pool to form candidate terms; these are listed below:
  (i) Calculate IG score.
  (ii) Calculate cooccurrence score.
  (iii) Calculate KLD score.
  (iv) Calculate RSV score.

Top scored candidate terms obtained from substeps (i) to (iv) of step (3) are used to expand the user query and called IGBQE, CBQE, KLDBQE, and RSVBQE, respectively.

 (4) Borda rank aggregation methods are used to combine different candidate term ranks obtained from substeps (i) to (iv) of step (3).
   (i) Borda rank aggregation produced a ranked list of candidate terms.

Some top *n* candidate terms obtained from substep (i) of step (4) are used to expand the user query and called BBQE.

 (5) Semantic filtering approach is used to filter out semantically irrelevant expansion terms from expansion terms set obtained from BBQE approach. After applying semantic filtering, this Borda and semantic based approach is called BSBQE.

### 3.2. Proposed Borda Based Query Expansion

After applying different query expansion terms selection methods, we got a separately ranked list of QE terms from each terms selection method. Now we need some rank combination approach that can combine different ranked lists of QE terms into a single list of terms. Now, some top scored terms are selected from this single list of terms as QE terms with the user query. In this section, we brief the reader about the ranks combination methods based on rank positions that we used in our proposed work. The social choice theory [21] is a study field in which voting algorithms are used as a technique for making the social or group decision. Algorithms used in this section are based on voting in the elections.

### 3.3. Borda Count Ranks Combining Approach

According to Borda ranks combining approach, each voter has their own preference list of candidates. For each voter, the top first candidate obtains *m* points, the top second candidate obtains *m* − 1 points, the top third candidate obtains *m* − 2 points, and so on. The sum value of obtained points of each voter gives the final points to each candidate. There are few candidates that are unranked by a voter (candidate terms selection method); then remaining points are divided among the unranked candidates. The candidate that has high points wins [22].

**Example 1 .**

Here we used an example to illustrate the working of Borda ranks combining approach. Here, we assume a combined single query expansion terms selection method with five following ranked query expansion terms selection methods, which ranked four candidate terms *P*, *Q*, *R*, and *S* as follows:

 Candidate terms selection method 1: *P*, *Q*, *R*, and *S*.
 Candidate terms selection method 2: *Q*, *P*, *S*, and *R*.
 Candidate terms selection method 3: *R*, *Q*, *P*, and *S*.
 Candidate terms selection method 4: *R*, *Q*, and *S*.
 Candidate terms selection method 5: *R*, *Q*.

Now we denote the score of each candidate term *t* by candidate score (*t*).

Borda ranking (for Example 1): the scores for each candidate term are as follows:

 Candidate score (*P*) = 4 + 3 + 2 + 1 + 1.5 = 11.5.
 Candidate score (*Q*) = 3 + 4 + 3 + 3 + 3 = 16.
 Candidate score (*R*) = 2 + 1 + 4 + 4 + 4 = 15.
 Candidate score (*S*) = 1 + 2 + 1 + 2 + 1.5 = 7.5.

Thus, the final ranking of candidate terms is *Q*, *R*, *P*, and *S*.

Some high ranked candidate terms selected by Borda scheme are used for expanding the user query: this type of QE is called Borda based query expansion (BBQE).

### 3.4. Proposed Semantic Filtering Based Query Expansion

A list of candidate terms is obtained after ranks combination modules. In this candidate terms list, we observed that some candidate terms as expansion terms are not related to the original user query. If we use these candidate terms as query expansion terms, it may retrieve irrelevant documents. Thus, it is compulsory to filter out these irrelevant candidate terms. To eliminate the irrelevant and redundant candidate expansion terms, we used the concept of semantic similarity that captures the semantically related terms with query terms from the candidate terms list and filters semantically nonrelated terms. For applying semantic similarity, we used linguistic ontology WordNet as background knowledge. The basic idea of semantic similarity is that if a candidate term has some kind of semantic relation (i.e., synonym, hypernym) with the query term then it will be appropriate for query expansion. According to the discussion in this section, there are a number of semantic similarity finding modules that can be used to find semantic similarity between two words or terms or concepts (such as query term and candidate term). The popular and feasible semantic similarity modules/approaches are Leacock and Chodorow (Lch) [23], Resnik [24], and Wu and Palmar [25], which takes two words/concepts as input and returns semantic similarity between these two terms. We used Leacock-Chodorow (Lch) semantic similarity measure in our work and found that results are motivating.

In this paper authors also have tried to handle sentiment and emotions to some extent, by using the approach present in [12]. For this purpose, first sentiment words are selected from the user query using background knowledge (SentiWordNet) and these words are expanded by adding other related sentiment and emotional words.

The Lch method defines a semantic similarity measure based on the shortest distance length (*c*_1_, *c*_2_) between two concepts or terms *c*_1_ and *c*_2_ and scaling that value by twice the maximum depth of the hierarchy, given in

(16) $$\begin{array}{c} {\text{Sim}}_{\text{lch}}\left(\;c_{1},c_{2}\;\right)=\max\left[\;-\log\left[\;\frac{\text{length}\left(c_{1},c_{2}\right)}{2\text{Dp}}\;\right]\;\right], \end{array}$$

where Dp is the maximum depth (i.e., 12 in case of WordNet-3.0); note that, in practice, we add 1 to both length (*c*_1_, *c*_2_) and 2Dp to avoid log⁡(0), when shortest path length is 0.

Our semantic filtering based approach BSBQE takes candidate terms as an input from BBQE approach and filters semantically irrelevant terms from the candidate terms list. We give a new formula for finding semantically similar expansion terms from candidate terms set. The new suggested formula is given in the following equation used to find semantic similarity between candidate term and the query terms:

(17) semantic  similarity  score  for  Q=SemSimc,Q=∑qi  in  QSimlchc,qi,

where *Q* is all query terms, *c* is a single candidate term, and *q*_*i*_ is an *i*th term of the query. Finally, noisy or irrelevant terms of BBQE are filtered by this semantic approach, and this semantic based approach is called BSBQE. The algorithmic steps of our proposed semantic based QE approach are listed in Algorithm 2.

**Algorithm 2 (developed for finding semantic similarity).**

 (1) Once the candidate terms sets are obtained from step (4) of Algorithm 1.
 (2) Input two terms/concepts *c*_1_ and *c*_2_; the first term *c*_1_ is obtained from step (1) and the second *c*_2_ is query term.
 (3) Words validation:
   If both words are present in English WordNet lexical taxonomy, go to step (4).
   Else, go to step (9).
 (4) Hypernym tree module: find hypernym tree of *c*_1_ and *c*_2_ using WordNet Taxonomy.
 (5) Hypernymy validation module: find if both trees have the same root or not.
    If root is the same, go to step (6).
   Else, go to step (9).
 (6) LCS module: find nearest common hypernym ancestor node of both words in the hypernym tree, which is called least common subsumer (LCS).
 (7) Count numbers of edges between *c*_1_ and *c*_2_ through LCS giving length (*c*_1_, *c*_2_). Then apply Lch semantic similarity measuring (16) method (note that Lch method uses max depth Dp for English WordNet version 3.0 taxonomy which is fixed and equal to 12).
 (8) Output numeric value of semantic similarity between two terms *c*_1_ and *c*_2_.
 (9) Stop.
 (10) Semantic similarity between candidate term and all query terms is obtained from (17).

### 3.5. Methods for Reweighting the Expanded Query Terms

After one of the QE terms selection methods described above has generated the list of candidate terms, the selected candidate terms that system adds to the user query must be reweighted. Different methods have been proposed for QE terms reweighting. We made a comparison analysis of these methods and tested which one is the most appropriate for our proposed AQE model. The most traditional and simple approach of expansion term reweighting is the Rocchio algorithm [26]. In this proposed work, we used Rocchio's beta version of Rocchio's algorithm, in which we require only the *β* parameter. Finally, we computed the new weight qtw of candidate terms used as expansion terms with the original user query as follows:

(18) $$\begin{array}{c} \text{qtw}=\frac{\text{qtf}}{{\text{qtf}}_{\max}}+\beta \frac{w\left(t\right)}{w_{\max}\left(t\right)}. \end{array}$$

In (18), parameter *w*(*t*) is the old weight of candidate term *t* and *w*_max_(*t*) is the maximum weight of the expanded query terms. *β* is a setting parameter, qtf is the query term *t* frequency, and qtf_max_ is the query term *t* maximum frequency present in the query *q*. The value of the parameter *β* is fixed to 0.1 in our experiment. Finally, the selected candidate terms are used after reweighting for expanding the user query.

## 4. Experimental Study

All the experiments carried out in this paper are based on the model proposed in Section 3. First, the performances of individual methods such as CBQE, RSVBQE, IGBQE, and KLDBQE are compared with each other or with Okapi-BM25 [18]. Second, the performance of BBQE and BSBQE is compared with Aguera and Araujo's model (combining multiple terms selection methods) [5] using different performance evaluation parameters.

### 4.1. Datasets

In this section, we describe two well-known benchmarks test collections used in our experiments: TREC disks 1 and 2 and FIRE ad hoc dataset, which are different in size and genre (TREC disks 1 and 2 size is 6 Gb, while FIRE dataset is 3.4 Gb). The detailed descriptions of both datasets are given in Table 1. Query numbers ranging from 126 to 175 are used for FIRE dataset and query numbers ranging from 151 to 200 are used for TREC dataset (a different collection of 50 queries is used for both datasets). The TREC disks 1 and 2 collections contain newswire article from different sources, such as Association Press, Wall Street Journal, Financial Times, and Federal Register, which are considered as high-quality text data with minimum noise. The FIRE ad hoc dataset is a medium size collection containing newswire article from two different sources named The Telegraph and BD News 24 provided by the Indian Statistical Institute, Kolkata, India.

In our experiments, we use only title field of TREC and FIRE query sets for retrieval task, because this field is closer to the actual queries used in real time applications. The last column of Table 1 presents the average documents length in the corresponding TREC and FIRE datasets.

Based on the performance, Porter stemmer is used to stem each term in the process of indexing and querying, and a latest list of 420 stop words is used to remove the stop words. In both FIRE and TREC dataset, the top 10, 25, and 50 retrieved documents are used to measure the average precision, recall, and mean average precision.

### 4.2. Parameter Tuning

To investigate the optimal setting of parameters for fair comparisons, we used the training method explained in Diaz and Metzler [27] for our proposed model, which is very popular in IR's field. First, for parameters in PRF models, we used different numbers of top feedback documents in both baseline and proposed approaches (5, 10, 15, 25, and 50), to find the optimal number of feedback documents. Here, we found that our proposed model performed best for top 15 numbers of feedback document; that is why we fix top 15 feedback documents to make the term pool in our experiment. Second, we select different number of top candidate terms from ranked candidate terms based on similarity value with query terms as expansion terms (10, 20, 30, 50, and 75), for both baseline and proposed methods to find the optimal number of top expansion terms used for reformulating query. Here, we found that our proposed model performed best for top 30 candidate terms; that is why we fix top 30 candidate terms to reformulate the original user query in our experiment.

### 4.3. Evaluation Parameters

Recall (*R*), precision (*P*), and *F*-measure are three parameters that are used to evaluate the performance of information retrieval system; recall is given by

(19) $$\begin{array}{c} \text{recall}=\frac{\left|R_{r}\right|}{\left|S_{\text{arel}}\right|}, \end{array}$$

where *R*_*r*_ is the set of relevant documents retrieved and *S*_arel_ is the set of all relevant documents, and

(20) $$\begin{array}{c} \text{precision}=\frac{\left|R_{r}\right|}{\left|S_{\text{ret}}\right|}, \end{array}$$

where *S*_ret_ is the retrieved documents set.

The average precision (AP) is used as a standard measure to find the quality of a search system in information retrieval. The precision of a document *d* is defined as the fraction of relevant documents within the set of retrieved documents. The AP for a relevant documents set is obtained as the mean precision of all these docs:

(21) $$\begin{array}{c} \text{AP}=\frac{1}{n}\overset{n}{\underset{i=1}{\sum }}\text{precision}\left(\;P_{i}\;\right), \end{array}$$

where *R*_*i*_ is the relevant documents set.

In general there has to be the trade-off between precision and recall as both of them cannot increase simultaneously. Depending on the requirement, we may be interested in higher precision or higher recall. However, if we want to evaluate the accuracy considering both precision and recall, we may use the *F*-measure to evaluate the accuracy of the result. The *F*-measure is a harmonic combination of the precision (*P*_*i*_) and recall (*R*_*i*_) values of the *i*th documents set used in information retrieval.

The *F*-measure can be calculated as follows:

(22) $$\begin{array}{c} F_{i}=\frac{2P_{i}R_{i}}{P_{i}+R_{i}}. \end{array}$$

We use these evaluation metrics as the primary single summary performance metric in our experiments that are also the main official evaluation metric in the corresponding TREC and FIRE evaluations forum. To make more confirm the superiority of our proposed method results, we used fixed-level interpolated precision-recall (the PR curve) curve for making the basic comparisons of our proposed method with other methods.

### 4.4. Experimental Results of Individual QE Terms Selection Methods

Tables 2 and 3 show the retrieval performance of query expansion terms selection methods in terms of average precision and recall on FIRE and TREC datasets and compared with Okapi-BM25 retrieval model, where Okapi-BM25 is a state-of-the-art probabilistic retrieval model [14].

In our experiment, we found that the performance of our proposed query expansion terms selection approaches IGBQE, CBQE, KLDBQE, and RSVBQE achieved a significant improvement over basic retrieval model Okapi-BM25. We also note that the improvements achieved by the proposed model on TREC disks 1 and 2 are little greater than the FIRE dataset. This is probably because the disks 1 and 2 collections contain news articles, which are usually considered as high-quality text data with less noise. On the contrary, FIRE ad hoc dataset contains news as well as web collections that are more challenging and include multiple sources of a heterogeneous set of the documents as well as more noise. Tables 2 and 3 show that the performance of KLD based query expansion terms selection method (KLDBQE) is higher than other terms selection methods in all top retrieved documents sets on both FIRE and TREC datasets.

Figure 2 shows the significant improvement by all used individual terms selection methods over Okapi-BM25 and the superiority of KLDBQE over other individual methods on both FIRE and TREC datasets.

**Figure 3:**
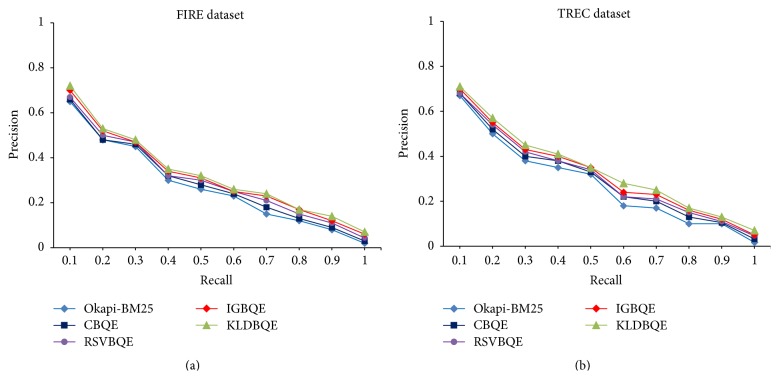
Precision-recall curve of all individual approaches on both FIRE and TREC dataset.

The 11-point precision-recall curves of all used individual terms selection methods, namely, CBQE, RSVBQE, IGBQE, and KLDBQE, with baseline approach Okapi-BM25 are shown in Figure 3. The 11-point precision-recall curve is a graph plotting the interpolated precision of an information retrieval (IR) system at 11 standard recall levels, that is, {0.0,0.1,0.2,…, 1.0}. The graph is widely used to evaluate IR systems that return ranked documents, which are common in modern search systems. Figure 3 also shows the significant improvement of individual terms selection approaches over baseline approach on both datasets. This also indicates the superiority of KLDBQE over other individual approaches.

### 4.5. Experimental Results of Borda and Semantic Similarity Methods

Tables 4 and 5 show the retrieval performance of our proposed Borda rank combination methods with or without semantic similarity in terms of average precision and recall on both FIRE and TREC datasets. Then, we compared the proposed model with Aguera and Araujo's model (model based on combining three QE terms selection methods) [5], where Aguera and Araujo's model is a state-of-the-art multiple QE terms selection combination based retrieval model.

Both Tables 4 and 5 also present the results of Okapi-BM25 and KLDBQE (best performing the individual method) methods for better comparisons. In our experiment, Tables 4 and 5 show that the performance of our proposed Borda based QE approach BBQE alone and with semantic similarity BSBQE achieved significant improvement over Okapi-BM25 model, KLDBQE (best individual QE terms selection method), and Aguera and Araujo's methods.

**Figure 4:**
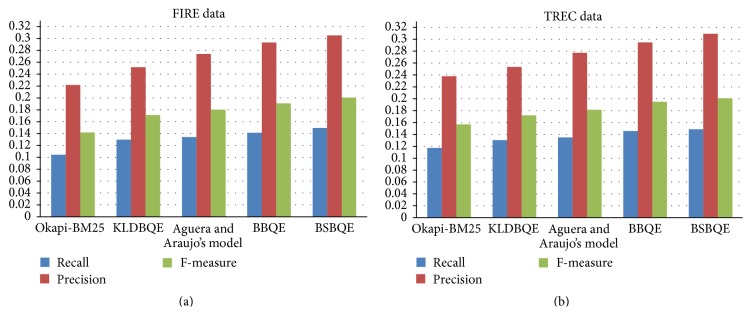
Recall, precision, and *F*-measures values of both proposed approaches on both FIRE and TREC dataset (for top 10 retrieved documents, discussed in Tables 4 and 5).

Figure 4 shows the significant improvement by our proposed BBQE and BSBQE over Okapi-BM25 and Aguera and Araujo's model in terms of recall, precision, and *F*-measures on both FIRE and TREC datasets.

**Figure 5:**
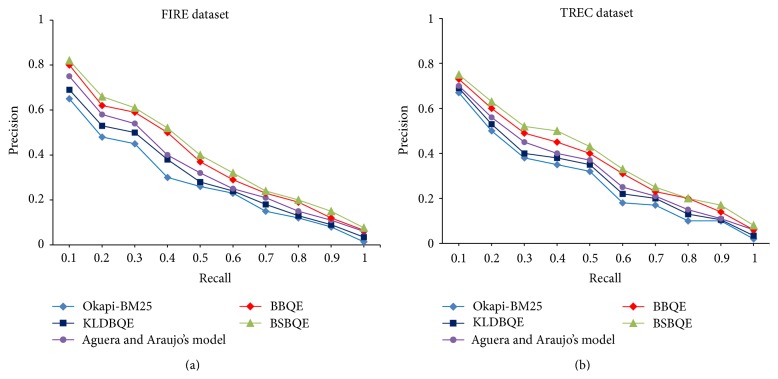
The precision-recall curve of both proposed approaches on both FIRE and TREC dataset.

The 11-point precision-recall curves of proposed approaches, namely, BBQE, BSBQE, and baseline approaches Okapi-BM25 and Aguera and Araujo's model are shown in Figure 5. The 11-point precision-recall curve is a graph plotting the interpolated precision of an information retrieval (IR) system at 11 standard recall levels, that is, {0.0,0.1,0.2,…, 1.0}. The graph is widely used to evaluate IR systems that return ranked documents, which are common in modern search systems. Figure 5 also shows the significant improvement of both our proposed approaches over baseline approaches. This indicates that the combination of both Borda rank aggregation scheme and semantic similarity scheme has the positive effect on improving the quality of expansion terms.

### 4.6. Statistical Analysis

#### 4.6.1. Statistical Significance of the Proposed Approaches

After observing that our proposed approach is giving better performance than the best of individual similarity measure considered, a *t*-test was applied to show that the improvement is statistically significant. This paired *t*-test compares one set of measurements with a second set from the same sample. Given two paired sets *X*_*i*_ and *Y*_*j*_ of *n* measured values, the paired *t*-test determines whether they differ from each other in a significant way under the assumptions that the paired differences are independent and identically normally distributed.

The statistical paired *t*-test results obtained for FIRE and TREC datasets are tabulated in Tables 6-7. A paired *t*-test is the most commonly used hypothesis test in IR. In the present work, the paired *t*-tests are conducted to determine whether the proposed query expansion approaches are statistically different from KLDBQE (best individual method) and Aguera and Araujo's model or not. These paired *t*-tests return the results in terms of *h*-value, *p* value, and CI values. The *p* value = 0 indicates that the null hypothesis is rejected and that the mean of our data is significantly different from other approaches with 95% certainty and therefore the null hypothesis “means are equal” cannot be rejected at the 5% significance level (*a* = 0.05).

If the *p* value = 1, then the performances are not statistically different and therefore the null hypothesis (“means are equal”) can be rejected at the 5% significance level (*a* = 0.05). CI is the 95% confidence interval of the mean based upon the *t*-distribution.  Table 6 clearly indicates that the improvement of the proposed Borda rank aggregating approaches over KLDBQE method is statistically significant at *a* = 0.05 (*p* is almost zero for both the FIRE and TREC dataset).

Table 7 shows paired *t*-test values between both our proposed approaches and Aguera and Araujo's model. The tables contain only the proposed approaches that pass the paired *t*-test. In our experiment, we compared both our proposed approaches with Aguera and Araujo's model. Table 7 clearly indicates that the improvement of our proposed approaches BBQE and BSBQE over Aguera and Araujo's model is statistically significant at *a* = 0.05 (*p* is almost zero for both the FIRE and TREC datasets).

### 4.7. Summary

Our observations on the experimental results of the query expansion score combination and rank combination of the query expansion selection methods are summarized as follows:

(i) The individual query expansion terms selection methods, namely, IGBQE, CBQE, RSVBQE, and KLDBQE, perform better than Okapi-BM25 (nonquery expansion method). In all used terms selection methods, KLDBQE performed best among CBQE, IGBQE, and RSVBQE.
(ii) Our proposed Borda based approach BBQE achieved motivational results and performed significantly better than the Okapi-BM25 model, KLDBQE (best individual expansion terms selection method), and Aguera and Araujo's method.
(iii) Our proposed Borda and semantic filtering approach BSBQE also performed better than the Okapi-BM25 model, KLDBQE (best individual expansion terms selection method), BBQE (best rank aggregation method), and Aguera and Araujo's method.
(iv) Paired *t*-test shows statistical significance of our proposed approaches over baseline approach in terms of *h*-value, *p* value, and CI value as shown in Tables 6-7.

## 5. Conclusion and Future Work

In this work, we explored the power of combining multiple query expansion terms selection methods to improve the performance of the information retrieval system by using AQE. We studied the Borda rank combination of four QE terms selection methods on two real datasets with or without semantic similarity approach. In our experiment, we observed that applying semantic similarity after Borda rank aggregations outperformed each individual QE terms selection method in terms of the average precision, recall, and *F*-measure values. In that case, different query expansion terms selection methods can capture the different characteristics of the terms, and the newly obtained terms can represent the documents set more accurately.

In this paper, we presented a new Borda rank combination based AQE method for document retrieval based on PRF techniques by mining additional QE terms, where our proposed Borda count based approach combines IG, cooccurrence, RSV, and KLD scores of candidate expansion terms and produces a single list of candidate expansion terms. The proposed Borda method uses voting approach to infer the weights of the additional query terms and then uses these additional query terms together with the original query terms to retrieve documents for improving the performance of information retrieval systems. After Borda approach, semantic similarity algorithms were used to filter out semantically irrelevant terms from candidate expansion terms obtained after Borda rank aggregation based query expansion approach.

TREC and FIRE benchmark datasets are used to validate our proposed QE method. The experiments confirmed that both our proposed QE methods increase the values of precision, recall, and *F*-measure. The higher values of average precision and average recall are also obtained by the proposed method in comparison to Okapi-BM25 and Aguera and Araujo's QE method. A paired *t*-test is conducted to present statistical analysis. This statistical analysis confirms that the proposed Borda based QE method significantly improves the IR efficiency as compared to Okapi-BM25 and Aguera and Araujo's approach. The robustness of the proposed QE model may be further tested on other TREC datasets.

## Figures and Tables

**Table 1:** Summary of used datasets and query numbers.

Datasets	Task	Queries	Docs	Number of unique terms	Average document length
TREC-3 (disks 1 and 2)	Ad hoc	151–200	7,41,856	14,83,71,200	349
FIRE	Ad hoc	126–175	4,56,329	6,27,56,468	273

**Table 2:** The comparison of different QE term selection methods with Okapi-BM25 in terms of average precision and recall for FIRE dataset.

Methods	Top 10 retrieved documents		Top 25 retrieved documents		Top 50 retrieved documents	
	Average precision	Average recall	Average precision	Average recall	Average precision	Average recall
Okapi-BM25	0.2217	0.1043	0.2175	0.1871	0.1839	0.2957
CBQE	0.2221	0.1176	0.2163	0.1965	0.1847	0.2963
RSVBQE	0.2380	0.1184	0.2201	0.1994	0.1895	0.3045
IGBQE	0.2419	0.1297	0.2458	0.2142	0.2391	0.3205
KLDBQE	0.2517	0.1299	0.2481	0.2150	0.2397	0.3268

**Table 3:** The comparison of different QE term selection methods with Okapi-BM25 in terms of average precision and recall for TREC dataset.

Methods	Top 10 retrieved documents		Top 25 retrieved documents		Top 50 retrieved documents	
	Average precision	Average recall	Average precision	Average recall	Average precision	Average recall
Okapi-BM25	0.2378	0.1172	0.2204	0.1911	0.1955	0.3012
CBQE	0.2381	0.1195	0.2216	0.1978	0.1990	0.3160
RSVBQE	0.2435	0.1193	0.2259	0.2096	0.2098	0.3159
IGBQE	0.2510	0.1236	0.2481	0.2147	0.2319	0.3271
KLDBQE	0.2536	0.1304	0.2594	0.2191	0.2413	0.3315

**Table 4:** Comparison of both our proposed approaches with Aguera and Araujo's model in terms of average precision and recall for FIRE dataset.

Methods	Top 10 retrieved documents		Top 25 retrieved documents		Top 50 retrieved documents	
	Average precision	Average recall	Average precision	Average recall	Average precision	Average recall
Okapi-BM25	0.2217	0.1043	0.2175	0.1871	0.1839	0.2957
KLDBQE	0.2517	0.1299	0.2481	0.2150	0.2397	0.3268
Aguera and Araujo's model	0.2738	0.1342	0.2545	0.2363	0.2510	0.3382
BBQE (proposed)	0.2932	0.1413	0.2769	0.2518	0.2656	0.3459
BSBQE (proposed)	0.3050	0.1494	0.2798	0.2612	0.2673	0.3506

**Table 5:** Comparison of both our proposed approaches with Aguera and Araujo's model in terms of average precision and recall for TREC dataset.

Methods	Top 10 retrieved documents		Top 25 retrieved documents		Top 50 retrieved documents	
	Average precision	Average recall	Average precision	Average recall	Average precision	Average recall
Okapi-BM25	0.2378	0.1172	0.2204	0.1911	0.1955	0.3012
KLDBQE	0.2536	0.1304	0.2594	0.2191	0.2413	0.3315
Aguera and Araujo's model	0.2774	0.1350	0.2581	0.2401	0.2593	0.3352
BBQE (proposed)	0.2948	0.1456	0.2795	0.2585	0.2696	0.3467
BSBQE (proposed)	0.3093	0.1487	0.2851	0.2657	0.2729	0.3544

**Table 6:** Paired *t*-test results between proposed approaches and KLDBQE approach for FIRE and TREC datasets.

Proposed approaches	Datasets	KLDBQE		
		*h*-value	*p* value	CI
BBQE	FIRE	1	0.0010	[−0.1813, −0.1104]
	TREC	1	0.0005	[−0.1033, −0.0763]

BSBQE	FIRE	1	0.0002	[−0.1738, −0.1040]
	TREC	1	0.0003	[−0.1672, −0.1160]

**Table 7:** Paired *t*-test results between both proposed approaches and Aguera and Araujo's model for FIRE and TREC datasets.

Proposed approaches	Datasets	Aguera and Araujo's model		
		*h*-value	*p* value	CI
BBQE	FIRE	1	0.0005	[−0.1712, −0.1010]
	TREC	1	0.0010	[−0.1137, −0.0694]

BSBQE	FIRE	1	0.0000	[−0.1610, −0.0842]
	TREC	1	0.0011	[−0.1385, −0.0473]

## Nomenclature

*Q*: Original user query
*D*: Set of initially retrieved documents by Okapi-BM25 function for user query
tf: Term frequency
qtf: Query term frequency
dl: Document length
avdl: Average document length
*N*: Number of documents in entire corpus
*T*: Set of some top initially retrieved documents for user query or set of top PRF docs
*C*: Entire corpus containing relevant and nonrelevant docs of user query
idf: Inverse document frequency
*N*_*C*_: Number of documents in the corpus that contain term *c*
SemSim(*c*, *Q*): Semantic similarity between a candidate term *c* and the query *Q*
Dp: Depth of WordNet or maximum depth of concepts
LCS: Least common subsume
Codegree(*q*_*i*_, *c*): Cooccurrence degree between a candidate term and the *i*th query term
Sim_lch_(*c*, *q*_*i*_): Lch module based semantic similarity between a candidate term and the *i*th query term
Tp: Term pool or candidate terms set containing all the unique terms of PRF docs
*c*: Single candidate term
docs: Documents.
